# Developing emotionally intelligent leaders within a chapter of a student pharmacist organization

**DOI:** 10.15694/mep.2018.0000262.1

**Published:** 2018-11-16

**Authors:** Erin Raney, Bill Bowman

**Affiliations:** 1 Midwestern University College of Pharmacy- Glendale

**Keywords:** emotional intelligence, leadership, student professional organization, pharmacy

## Abstract

This article was migrated. The article was marked as recommended.

**Introduction:** Emotional intelligence (EI) refers to an individual’s awareness, management, and use of emotions, which has been shown to correlate with successful leadership. The objective of this study was to facilitate the development of emotionally intelligent leaders amongst the student officers within a chapter of the American Pharmacists Association-Academy of Student Pharmacists.

**Methods:** During the 2012-2015 academic years, the chapter officers (n=27) participated in a leadership development program that included self-assessments, group discussions, reflection activities, and/or individual mentoring sessions based upon the concepts of organizational management, strengths-based leadership, team dysfunction, and continuous professional development. The officers also completed the Emotional Intelligence Appraisal® at the beginning and end of the program, and a perception survey after completing the program.

**Results:** The officers’ initial and final EI scores ranged from 54-100 and 59-100, respectively. In addition, their final mean overall, self-awareness, self-management, and relationship-management scores were higher than their corresponding initial scores ( *P* < 0.05). Most officers (89-100%) also rated each of their developmental experiences as being either very beneficial or beneficial.

**Conclusion:** The implemented program appears to be an effective method for increasing the EI of student organization officers and provides a model for similar efforts in other health professions settings.

## Introduction

Within the context of pharmacy education, leadership has been formally described as “the graduate being able to demonstrate responsibility for creating and achieving shared goals, regardless of position” ( ACPE, 2015; Medina *et al*., 2013). This description is reflective of an expanded emphasis upon the need to develop the leadership skills of all student pharmacists, which is in part attributed to a perceived leadership gap within the pharmacy profession ( Bradley-Baker and Murphy, 2013; Kerr *et al*., 2009; Zellmer, 2008; White, 2005; Holdford, 2003). Consequently, several publications have recently described best practices, guiding principles, and competencies for the deliberate integration of student leadership development into Doctor of Pharmacy programs ( Janke, Traynor and Boyle, 2013; Janke *et al*., 2016; Ross *et al*., 2013; Traynor, Boyle and Janke, 2013).

A cataloging of the strategies used by U.S. pharmacy programs for developing the leadership skills of student pharmacists has also been recently reported ( Feller, Doucette and Witry, 2016). Specific examples include seminar series, elective didactic coursework, co-curricular programming, and an extracurricular retreat. As faculty advisors for the American Pharmacists Association-Academy of Student Pharmacists (APhA-ASP) chapter at Midwestern University College of Pharmacy – Glendale (MWU-CPG), we previously reported on the pilot implementation of a leadership development strategy for our chapter officers ( Bowman et al. 2016). This strategy was created to offset the accelerated nature of MWU-CPG’s three-year PharmD curriculum, which is implemented through a year-round, four-quarter system (10 weeks each), and was shown to successfully expand upon the officers’ traditional duties. These results ultimately established a foundation for the implementation of a broader leadership development program based upon the concept of emotional intelligence (EI).

While the term “social intelligence” first emerged in the early 1900s, the formal introduction of the term “emotional intelligence” did not occur until the 1990s ( Salovey and Mayor, 1990; Goleman, 1995). Although definitions of EI vary widely, the term generally refers to an individual’s awareness, management, and use of emotions. In particular, EI has been described as “the ability to monitor one’s own and others’ feelings and emotions, to discriminate among them, and to use this information to guide one’s thinking and actions” ( Salovey and Mayor, 1990). Multiple models for evaluating and quantifying EI exist and each varies depending upon the underlying definitions utilized. Some key examples include the Mayer-Salovey-Caruso Emotional Intelligence Test (MSCEIT), Bar-On EQ-I, and Emotional Intelligence Appraisal® (EIA, *Emotional Intelligence 2.0*) ( MHS Assessments, 2018; Bradberry and Greaves, 2009). Within *Emotional Intelligence 2.0*, Bradberry and Greaves (2009) describe EI as “your ability to accurately perceive your own emotions in the moment and understand your tendencies across situations” and comprise it of both personal competence (self-awareness and self-management skills) and social competence (social awareness and relationship management skills).

Potential applications of EI have focused primarily upon the workplace, particularly employee productivity and job satisfaction ( Goleman, 1998). Additional applications within health professions and pharmacy training have been directed towards teaching professionalism, providing patient care, and building provider-patient relationships ( Lust and Moore, 2006; Romanelli, Cain and Smith, 2006; Taylor, Farver and Stoller, 2011). Like others, we also believe EI is an integral part of effective leadership ( Gallagher and Costal, 2012; Haight *et al*., 2017; Hall *et al*., 2015; Holdford, 2003; Nelson *et al*., 2015). Therefore, this report describes the implementation and evaluation of our broader effort to facilitate the development of emotionally intelligent leaders amongst the students serving as officers within our APhA-ASP chapter.

## Methods

### Program Implementation

Our leadership development program consisted of a series of voluntary activities, which were offered to 3 groups of APhA-ASP chapter officers (27 students total) as they completed their elected terms during the 2012-2015 academic years, respectively ( Table 1).

**Table 1.** Program to facilitate the development of emotionally intelligent leaders amongst the students serving as officers within our APhA-ASP Chapter

2 ^nd^ Didactic Year ^a^			
Fall Quarter	Winter Quarter	Spring Quarter	Summer Quarter
Participation in a group discussion regarding organizational management	Completion of the StrengthsFinder 2.0 Assessment	Completion of the Five Dysfunctions of a Team Assessment	Final completion of the EIA
Initial completion of the EIA	Participation in a group discussion regarding Strengths Based Leadership and the StrengthsFinder 2.0 Assessment	Participation in a group discussion regarding The Five Dysfunctions of a Team and the Team Assessment	Participation in a group discussion regarding the final EIA and continuous professional development
Completion of a personal goals self-reflection activity	Completion of a Strengths Based Leadership self-reflection activity	Participation in an individual mentoring session regarding the Team Assessment	Completion of a Continuous Professional Development portfolio activity
Participation in an individual mentoring session regarding the initial EIA and the personal goals self-reflection activity	Participation in an individual mentoring session regarding the Strengths Based Leadership self-reflection activity		Participation in an individual mentoring session regarding the final EIA and the Continuous Professional Development portfolio activity

^a^ the officers’ elected terms take place during their second didactic year, beginning at the start of the fall quarter and concluding at the end of the summer quarter

Each group began by exploring the concept of organizational management and completing an initial EIA, which asks users to respond to 28 different online survey questions by indicating how frequently they demonstrate a certain behavior. Subsequently, the officers explored the concepts of *Strengths Based Leadership* and *The Five Dysfunctions of a Team* to further facilitate the development of their personal and social competencies, and to examine the interplay between these competencies ( Rath and Conchie, 2008; Lencioni, 2002). The program then culminated with the officers completing a final EIA and exploring the concept of continuous professional development (ACPE, 2018). Each of the previous concepts was the focus of a separate academic quarter and was explored through a self-assessment, group discussion, reflection activity, and/or individual mentoring session while the officers were actively engaged in their respective leadership duties.

### Emotional intelligence scores

Actual skill development during the program was determined by comparing each chapter officer’s initial and final EIA results. Each officer’s results report provided an overall EI score along with scores for each of the four *Emotional Intelligence 2.0* skills (i.e. self-awareness, self-management, social awareness, and relationship management). These scores were established on a scale of 0-100, derived from a “normed” sample, and associated with a particular skill level. The officers’ resulting scores were analyzed using GraphPad Prism 6 for Windows, Version 6.05 and statistical analysis was conducted using paired, two-tailed, *t* tests (GraphPad Software, Inc., La Jolla, CA).

### Student perceptions

The chapter officers’ perceptions of their development during the program were determined using a survey instrument similar to the one created during our pilot implementation ( Bowman and Raney, 2016). Upon the conclusion of their elected terms, each officer was invited to voluntarily and anonymously complete the instrument via SurveyMonkey.com ( SurveyMonkey.com, Inc., Palo Alto, CA). The instrument asked the officers to indicate the extent to which they agreed with the statement “serving as an APhA-ASP officer facilitated my professional development” using a four-point Likert scale. The officers were also asked to indicate how beneficial each of a variety of listed APhA-ASP officer experiences was to their development (i.e. very beneficial, beneficial, somewhat beneficial, not beneficial); the officers could also indicate if a particular experience was not applicable. The instrument also included several open-ended questions that asked the officers to indicate which of the listed experiences was most beneficial, what additional APhA-ASP officer experiences were beneficial, and how may the APhA-ASP officer experience be improved. The collected data were analyzed in aggregate using descriptive statistics within Microsoft Office Excel 2010, Version 14 (Microsoft, Inc., Redmond, Washington). The MWU-Glendale Institutional Review Board found that this study fulfilled the criteria for exempt review.

## Results/Analysis

All of the chapter officers completed the leadership development program and the overall response rates for both the EIA and the perception survey were 100% (n=27). The officers’ initial and final individual EI scores ranged from 54-100 and 59-100, respectively, while their mean scores ranged from 74-79 and 79-82, respectively ( Table 2).

**Table 2.** Summary of the chapter officers’ EI scores

	*Initial EI Scores* ^a^				
	Overall	Self Awareness	Self Management	Social Awareness	Relationship Management
Mean(±SD)	76(±8) ^b^	75(±9) ^b^	74(±9) ^b^	79(±11)	76(±9) ^b^
Maximum	93	88	91	100	95
Minimum	64	60	57	54	63

	*Final EI Scores* ^a^				
	Overall	Self Awareness	Self Management	Social Awareness	Relationship Management
Mean(±SD)	80(±8) ^b^	82(±11) ^b^	79(±9) ^b^	81(±10)	79(±11) ^b^
Maximum	96	98	95	100	95
Minimum	66	63	59	61	65


^a^ n=27;

^b^ statistical difference ( *P* < 0.05) between initial and final EI scores

In addition, the officers’ final mean overall, self-awareness, self-management, and relationship management scores were higher than their corresponding initial scores ( *P* < 0.05), with the largest numerical increase being in self-awareness. However, there was no change in the officers’ mean social awareness scores ( *P* > 0.05).

All of the chapter officers either strongly agreed (n=24) or agreed (n=3) that serving as an APhA-ASP officer facilitated their development. Nearly all of the officers (96-100%) indicated that each of the listed APhA-ASP officer experiences ( Table 3) was applicable to their development, except for “attending any leadership development workshops at the Mid-Year Regional and/or Annual Meetings” (66%) and “attending the Summer Leadership Institute (SLI)” (26%), which are experiences that not all officers participate within.

**Table 3.** The mean level of benefit and frequency of being provided as most beneficial for each of the listed APhA-ASP officer experiences (n=27)

APhA-ASP Officer Experience	Mean Level of Benefit ^a^ (±SD)	Number of Most Beneficial Comments ^b^
Participating in the initial group discussion on organizational management	2.7±0.6	0
Completing the duties of your elected position	2.8±0.4	3
Attending any leadership development workshops at the Mid-Year Regional and/or Annual Meetings	2.4±0.8	0
Attending the Summer Leadership Institute (SLI)	2.9±0.4	1
Completing and individually discussing your initial Emotional Intelligence Appraisal with the chapter advisors	2.5±0.6	9
Completing and individually discussing the initial Development Goals Reflection Activity with the chapter advisors	2.5±0.6	6
Completing and individually discussing the Strength-Based Leadership activities with the chapter advisors	2.7±0.7	9
Completing and individually discussing the (Dys)functions of a Team activities with the chapter advisors	2.6±0.5	5
Completing and individually discussing your second Emotional Intelligence Appraisal with the chapter advisors	2.6±0.7	8
Completing and individually discussing the Continuous Professional Development activities with the chapter advisors	2.7±0.6	9
Participating in the group discussion on Strengths-Based Leadership	2.5±0.6	4
Participating in the group discussion on (Dys)functions of a Team	2.5±0.5	0
Participating in the group discussion regarding the Emotional Intelligence Appraisal results	2.5±0.6	3
Participating in the group discussion on Continuous Professional Development	2.5±0.6	2

^a^ calculated by assigning a numerical value to each of the applicable response options (ie. very beneficial=3, beneficial=2, somewhat beneficial=1, and not beneficial=0);

^b^ many officers provided multiple comments and/or comments that included multiple experiences, all of which were tallied

A mean level of benefit was also calculated for each of the listed experiences by assigning a numerical value to each of the applicable response options (ie. very beneficial=3, beneficial=2, somewhat beneficial=1, and not beneficial=0). The experiences resulting in the highest mean level of benefit were “attending the Summer Leadership Institute (SLI)” (2.9±0.4) and “completing the duties of your elected position” (2.8±0.4), while the experience resulting in the lowest mean level of benefit was “attending any leadership development workshops at the Mid-Year Regional and/or Annual Meetings” (2.4±0.8). In addition, the experiences most frequently provided as being most beneficial were “completing and individually discussing your initial Emotional Intelligence Appraisal / the Strength-Based Leadership activities / your second Emotional Intelligence Appraisal / the Continuous Professional Development activities with the chapter advisors” (n=8-9 each).

The comments provided for “what additional APhA-ASP officer experiences were beneficial” (n=22) and “how may the APhA-ASP officer experience be improved” (n=19) were tallied based upon each comment’s relevance to an identified theme, which enabled the resulting themes to be quantitatively ranked based upon the frequency of their respective comments. “Working together as a team” (n=6) and “informal interactions with the chapter advisors” (n=3) were the most prevalent themes identified amongst the additional experiences the officers found beneficial. The most prevalent theme identified for improving the officer experience was “providing additional / earlier team-building activities” (n=5).

## Discussion

The described program is purposeful in its use of EI to not only facilitate, but also assess, the leadership development of the students serving as officers within MWU-CPG’s APhA-ASP chapter. In addition, the program’s use of a variety of developmental tools and frequent intervention formats also targets each component of EI in a longitudinal fashion, a recommendation supported by Feller et al. in their review of leadership development opportunities in U.S. Schools of Pharmacy ( Feller, Doucette and Witry, 2016). In our experience, optimizing the level of student engagement within such activities can be challenging and therefore a variety of active learning approaches were utilized and the overall value of the students’ participation was ensured. Integrating the program with the traditional duties of the chapter officers also enabled each of the explored leadership concepts to be immediately and directly applied in an experiential fashion.

The results of this study indicate that during the leadership development program the chapter officer’s self-awareness, self-management, and relationship management skills collectively improved. Overall, the degree of these changes represents an advancement in skill from a level described by Bradberry and Greaves as “ *with a little improvement, this could be a strength*” to a level described as “ *a strength to build on*” (2009). In addition, the fact that the largest numerical increase was in the officers’ mean self-awareness score seems reasonable, as this is the most foundational EI skill. Smith et al also reported on the changes in EI scores for 38 pharmacy students who completed a 3-year leadership degree option program at the University of Oklahoma ( Smith *et al*., 2018). They similarly found a statistically significant increase in the students’ mean overall score (pre = 76.39, post = 81.03; p 

The chapter officers’ perceptions also indicate that serving as an APhA-ASP officer had a very positive impact upon their professional development. In addition, there is overwhelming evidence that each component of our leadership program was perceived as being beneficial to the officers’ development, with the individual mentoring sessions being perceived most positively. This particular perception is likely due to the fact that these sessions enabled us to provide very individualized feedback on each of the officers’ greatest concerns. The responses to the ‘what additional experiences’ and ‘how may the experience be improved’ questions also demonstrate that the officers placed great value upon the concept of teamwork. Overall, the results of this study indicate that the described program may be an effective method for facilitating the development of emotionally intelligent leaders within a chapter of a professional student organization.

In general, our leadership program addresses many of the challenges faced in facilitating such development in student pharmacists, particularly within the time constraints of an accelerated, 3-year curriculum, and appears to have enhanced the overall value of serving as a chapter officer within the APhA-ASP. However, while the findings of this study are reflective of several groups of students over time, they are limited to the experiences of a relatively small number of students within one student organization at a single College of Pharmacy. We also recognize that while the components of our program may have contributed to the development of EI, each chapter officer had additional professional and personal experiences during the time of this study that likely influenced their EI scoring as well. In addition, while we agree that the key components of EI are integral parts of effective leadership, EI may not actually be the best measure of such development.

The EI assessment tool chosen for this study is an additional consideration. There are many tools available for the measurement of EI, each with different scales and descriptors. We recognize that any interpretation of EI change is limited to the construct of each tool and is not directly comparable across all EI assessments. Our choice of the EIA was based upon 1) the assessment’s correlation with the job performance of those in leadership positions, 2) the option to use the *Emotional Intelligence 2.0* book and corresponding results report as supplements for EI development, 3) the ability to complete the assessment twice for a pre- and post-comparison, and 4) *Emotional Intelligence 2.0’s* relative affordability and accessibility. In addition, *Emotional Intelligence 2.0* has been widely adopted and is a quick and intuitive approach to EI, which ultimately seemed to fit with the culture of our institution. While we are pleased with the integration of the EIA into the structure of our leadership program, we do appreciate that this assessment is based solely upon self-perceptions and thus does not provide an opportunity for outside perspectives, which would provide a more robust view of EI development.

The long-term goal of this work is to further elucidate how leadership concepts may be best incorporated into contemporary health professions programs and how to best develop the leadership skills of all students within the contexts of our unique institutions. Overall, we believe the described program successfully built upon our initial pilot strategy and provides valuable insight for others attempting to develop the leadership skills of health professions students and/or close any perceived leadership gaps within the health professions. For example, in their recommendations for incorporating EI into medical education, Roth et al. highlighted the importance of relating EI concepts to leadership development, specifically, in support of the broader trend within the medical profession ( Roth *et al*., 2018). With regard to our own efforts, this study has provided sufficient evidence to enable the expansion of our program to include all of the officers within MWU-CPG’s student organizations. Measurement of the impact of this recently implemented program upon EI is currently under investigation and will be reported on in the near future.

## Conclusion

The described program is purposeful in its use of EI to not only facilitate, but also assess, the leadership development of the students serving as officers within an APhA-ASP chapter. The chapter officers’ EI did significantly improve during the program. The chapter officers also perceived that the program had a very positive impact upon their development. Overall, this study provides valuable insight for others seeking to facilitate leadership development in health professions students and has provided sufficient evidence to expand our own efforts to include all of our College’s student organization officers.

## Take Home Messages

• Recognition of the need to develop the leadership skills of student pharmacists has been rapidly expanding, which is in part attributed to a perceived leadership gap within the pharmacy profession.
• Emotional intelligence is increasingly recognized as a significant contributor to successful leadership.
• A longitudinal leadership development program was created to provide opportunities for enhancing the emotional intelligence of student pharmacists in the areas of self-awareness, self-management, social awareness and relationship management.
• The described program appears to be an effective method for facilitating the development of emotionally intelligent leaders and offers a model for the expansion to other health professions settings.

## Notes On Contributors

• Erin Raney, PharmD, is a Professor of Pharmacy Practice at Midwestern University College of Pharmacy-Glendale.
• Bill Bowman, PhD, is an Associate Professor of Pharmaceutical Sciences at Midwestern University College of Pharmacy-Glendale.
