# Supplementary material for: Developing emotionally intelligent leaders within a chapter of a student pharmacist organization
Source: MedEdPublish (2016). 2018 Nov 16;7:262. [Version 1] doi: 10.15694/mep.2018.0000262.1 (PMC10898549; doi:10.15694/mep.2018.0000262.1)
Supplement: Peer review history

# Reviewer report: Reviewer response for version 1

**DOI:** 10.21956/mep.19722.r29530

**Published:** 2018-12-27

**Authors:** Judy McKimm

**Affiliations:** 1 Swansea University

This review has been migrated. The reviewer awarded 3 stars out of 5 I like that you've done a study on EI in Pharmacy as pharmacy leadership is less well developed than, say doctor or nurse leadership, so it's a welcome addition to the literature. The small self selected sample size is a limitation of course and so you can't draw too much of significance from this, but it would provide a useful spring board to a more systematic study. I seem to be missing something but what was the actual leadership development intervention and what and how was it designed to develop EI? I see the topics and activities but not how these are supposed to actually develop the EI. You might want to look at Petrie's (2014) work on vertical and horizontal leadership as some of the ways of developing EI (and associated qualities such as resilience and grit) relate to experiential learning and guided reflection. Nevertheless a good summary of the literature on pharmacy leadership.

Reviewer Expertise:

NA

No decision status is available

# Reviewer report: Reviewer response for version 1

**DOI:** 10.21956/mep.19722.r29528

**Published:** 2018-11-25

**Authors:** Ken Masters

**Affiliations:** 1 Sultan Qaboos University

This review has been migrated. The reviewer awarded 3 stars out of 5 A well written paper, solidly grounded in the literature from both the EI quarter and pharmacy leadership.Although the results are encouraging, it would be useful if the authors could draw readers’ attention to the limitations. Most notably is the very small sample size. This makes statistical analyses difficult, and generalizability tenuous. In addition, although there are statistically significant differences in most of the means, if one looks closely at all of the figures, one sees that the improvements are small (although expected with the figures in the 90s), and one would have to wonder about the real-world impact of these differences. (Much like the relationship between statistically significant, and clinically significant differences). For this impact to be more clearly understood, it would have been useful if the authors could offer some insight into the impact of these figures, perhaps from the literature or from their own experience.Nevertheless, the paper is a good a useful read.

Reviewer Expertise:

NA

No decision status is available

# Reviewer report: Reviewer response for version 1

**DOI:** 10.21956/mep.19722.r29531

**Published:** 2018-11-23

**Authors:** Ariela Marshall

**Affiliations:** 1 Mayo Clinic

This review has been migrated. The reviewer awarded 4 stars out of 5 This is an excellent description of a leadership development program designed to increase participants’ Emotional Intelligence (EI). EI is an essential leadership skill and I commend the authors on recognizing its importance and developing what appears to be a successful strategy to improve skills in this area. I would like to hear a bit more about the following points:(1) What specific leadership skills are useful for pharmacy students/pharmacists, and how do the authors envision the role of the pharmacist leader? The introduction discusses a “perceived leadership gap within the pharmacy profession” and there is good discussion of the general principles of leadership but I would like to hear more about the specific role of the pharmacist leader and what this entails. Do the authors see pharmacists taking clinical leadership roles on an inpatient/outpatient team? Administrative leadership roles at a pharmaceutical company? It would be good to provide specific examples of leadership potential for pharmacists and how increasing EI may help individuals achieve these positions/goals(2) Election to the role of an officer in a national organization and participation in this group may itself improve leadership skills and perceived EI. It would be helpful for the authors to discuss how they could separate out the specific contribution of this leadership development program to the improvement in EI versus the improvement that may be seen over the course of a year simply by students who perceive themselves and who are perceived by their peers as leaders by serving as officers in this organization. It would be good to have a comparison group (i.e. historical “control” group of students who took the EI test at the start/completion of the year) to see if there were changes in this cohort who did not participate in the leadership program just by virtue of serving as an officer in the organization. If there were no changes in EI in a control group, that would strengthen the claim that it was the leadership development program specifically which helped increase EI.

Reviewer Expertise:

NA

No decision status is available

# Reviewer report: Reviewer response for version 1

**DOI:** 10.21956/mep.19722.r29529

**Published:** 2018-11-20

**Authors:** Mary Anne Cordero

**Affiliations:** 1 Princess Nourah Bint Abdulrahman University

This review has been migrated. The reviewer awarded 4 stars out of 5 No doubt that Emotional Intelligence is an integral part of effective leadership as mentioned in the introduction. I think it’s also worth elucidating the clinical or practical applications and significance of this leadership program for future practice in health profession. Citing relevant scholarships related to this is commendable. The topics and activities included in the leadership program were listed in table 1 but I think it’s also worthy to note the program details like how sessions were conducted, teaching and learning strategies employed, faculty/facilitators involved, the time allotted for each session, and the setting. In the results, no significant difference was observed between participants’ initial and final mean scores under social awareness component. This may be discussed in the paper with impetus on how the aspect of social component may be addressed or improved. Barriers and difficulties may be cited in the programs’ evaluation or revision prior to its replication in larger population of students.

Reviewer Expertise:

NA

No decision status is available

# Reviewer report: Reviewer response for version 1

**DOI:** 10.21956/mep.19722.r29532

**Published:** 2018-11-17

**Authors:** Balakrishnan Nair

**Affiliations:** 1 Centre for Medical Professional Development and University of Newcastle

This review has been migrated. The reviewer awarded 3 stars out of 5 All health care professional students should be taught leadership skills ; emotional intelligence is an essential part of leadership. All good leaders have EI. This study attempted to teach EI and evaluate the outcome on pharmacy students according to the title . In the Methods section , it is described as EI teaching to “officers” . I am not sure what is the difference between students and officers and what the roles of the officers are In the evaluation , it is stated there is statistically significant improvement in the outcome; however is this “clinically “ significant ? Some discussion would be useful for the readership

Reviewer Expertise:

NA

No decision status is available
